# Confirmation of the *OVOL2* Promoter Mutation c.-307T>C in Posterior Polymorphous Corneal Dystrophy 1

**DOI:** 10.1371/journal.pone.0169215

**Published:** 2017-01-03

**Authors:** Doug D. Chung, Ricardo F. Frausto, Aleck E. Cervantes, Katherine M. Gee, Marina Zakharevich, Evelyn M. Hanser, Edwin M. Stone, Elise Heon, Anthony J. Aldave

**Affiliations:** 1 Stein Eye Institute, David Geffen School of Medicine at the University of California Los Angeles, Los Angeles, California, United States of America; 2 Department of Ophthalmology, University of Iowa Carver College of Medicine, Iowa City, Iowa, United States of America; 3 Department of Ophthalmology and Vision Sciences, The Hospital for Sick Children, University of Toronto, Toronto, Ontario, Canada; Medizinische Universitat Innsbruck Department fur Kinder- und Jugendheilkunde, AUSTRIA

## Abstract

**Purpose:**

To identify the genetic basis of posterior polymorphous corneal dystrophy (PPCD) in families mapped to the PPCD1 locus and in affected individuals without *ZEB1* coding region mutations.

**Methods:**

The promoter, 5’ UTR, and coding regions of *OVOL2* was screened in the PPCD family in which linkage analysis established the PPCD1 locus and in 26 PPCD probands who did not harbor a *ZEB1* mutation. Copy number variation (CNV) analysis in the PPCD1 and PPCD3 intervals was performed on DNA samples from eight probands using aCGH. Luciferase reporter assays were performed in human corneal endothelial cells to determine the impact of the identified potentially pathogenic variants on *OVOL2* promoter activity.

**Results:**

*OVOL2* mutation analysis in the first PPCD1-linked family demonstrated segregation of the c.-307T>C variant with the affected phenotype. In the other 26 probands screened, one heterozygous coding region variant and five promoter region heterozygous variants were identified, though none are likely pathogenic based on allele frequency. Array CGH in the PPCD1 and PPCD3 loci excluded the presence of CNV involving either *OVOL2* or *ZEB1*, respectively. The c.-307T>C variant demonstrated increased promoter activity in corneal endothelial cells when compared to the wild-type sequence as has been demonstrated previously in another cell type.

**Conclusions:**

Previously identified as the cause of PPCD1, the *OVOL2* promoter variant c.-307T>C was herein identified in the original family that established the PPCD1 locus. However, the failure to identify presumed pathogenic coding or non-coding *OVOL2* or *ZEB1* variants, or CNV involving the PPCD1 and PPCD3 loci in 26 other PPCD probands suggests that other genetic loci may be involved in the pathogenesis of PPCD.

## Introduction

Posterior polymorphous corneal dystrophy (PPCD) is an autosomal dominant disorder characterized by the presence of characteristic corneal endothelial opacities as well as corneal steepening. While the majority of affected individuals are thought to be asymptomatic, the percentage of individuals who require corneal transplantation for corneal edema is estimated to be approximately 20%-25%, significantly higher than previously believed.[1] PPCD demonstrates genetic heterogeneity, having been mapped to both chromosome 10 (PPCD3 locus) and 20 (PPCD1 locus). To date, approximately 30% to 40% of PPCD cases have been attributed to 40 unique heterozygous nonsense and frameshift mutations and hemizygous copy number variants involving the zinc finger E-box binding homeobox 1 gene (*ZEB1*) on chromosome 10 that likely result in *ZEB1* haploinsufficiency.[2–12] However, the identification of the genetic basis of PPCD1 (MIM ID #122000) has proven to be more elusive, in spite of the fact that linkage to the PPCD1 locus was reported almost a decade before linkage to the PPCD3 locus was described.[13, 14]

A 3.6 cM (1.8 Mb) interval is shared between four PPCD families that demonstrated linkage to the pericentromeric region of chromosome 20.[13, 15–20] However, mutational analysis of the coding sequence and exon-intron boundaries of the 32 positional candidate genes from the shared interval failed to identify a pathogenic mutation in one of the four families linked to the PPCD1 locus.[15–17, 19–23] Given this, we employed a targeted next-generation sequencing approach to identify potentially pathogenic non-coding variants in these 32 positional gene candidates. We identified 11 single-nucleotide variants (SNV) in nine genes that met our criteria of possible pathogenicity. Four of the SNVs were located in three different protein coding genes, three of which (*OVOL2* c.327C>A, *OVOL2* c.-307T>C, and *THBD* c.351A>G) segregated with the affected status when additional family members who did not undergo NGS were screened. Of these, only c.-307T>C, in the promoter region of the ovo-like zinc finger 2 (*OVOL2*) gene, was novel, and was not identified in 100 controls. [24] Davidson and colleagues recently reported the identification of c.-307T>C and two other novel *OVOL2* promoter region variants (c.-274T>G and c.-370T>C) in two British and 16 Czech PPCD families, which include two of the four families that have been previously mapped to the PPCD1 locus.[25] In addition, another *OVOL2* promoter region variant (c.-339_361dup) was identified in a family originally reported with autosomal dominant congenital hereditary endothelial dystrophy (CHED) mapped to the CHED1 locus, which overlaps the PPCD1 locus.[25] OVOL2 is a transcription factor that has been shown to downregulate ZEB1 expression, thereby suppressing EMT and driving mesenchymal-to-epithelial transition (MET) instead.[26, 27] Reporter activity assays using *OVOL2* promoter constructs each containing one of the four identified variants demonstrated increased *in vitro* reporter gene expression compared to the wild type promoter in HEK293 cells.[25] Thus, Davidson and colleagues proposed that PPCD-associated *OVOL2* promoter variants cause increased *OVOL2* expression and the encoded protein subsequently represses *ZEB1* expression, leading to a PPCD phenotype similar to PPCD3, in which *ZEB1* truncating mutations likely result in *ZEB1* haploinsufficiency.[4, 12]

Herein, we present the results of sequencing the 5’ UTR and promoter region of *OVOL2* in the family originally linked to the PPCD1 locus on chromosome 20, as well as in 26 PPCD probands in whom a *ZEB1* truncating mutation was not identified.[13] We also report the results of copy number variation (CNV) analysis in the PPCD1 and PPCD3 loci to identify the cause of PPCD in individuals without either an *OVOL2* or a *ZEB1* 5’ UTR, promoter, or coding region mutation. In addition, we present the results of *in vitro* promoter assays in human corneal endothelial cells to confirm the functional impact of the pathogenic *OVOL2* promoter variant in the affected cell type.

## Materials and Methods

This study followed the Declaration of Helsinki and was approved by the Institutional Review Board at the University of California at Los Angeles (UCLA IRB# 94-07-243-(14-33A), 02-10-092-(4,11),11–000020). Informed written consent was obtained from all subjects in this study.

### Patient selection and DNA collection

Individuals with PPCD were examined with slit lamp biomicroscopic imaging and the diagnosis of PPCD was made based upon previously described clinical characteristics.[3] After informed consent was obtained, genomic DNA was purified from peripheral blood leukocytes using the FlexiGene DNA Isolation Kit (Qiagen, Valencia, CA) or extracted from buccal epithelial cells using the Oragene Saliva Collection Kit (DNA Genotek, Ottawa, Canada) according to the manufacturer’s instructions.

### *ZEB1*—Polymerase chain reaction (PCR) and Sanger sequencing

Each of the nine exons of *ZEB1*, including an alternative exon 1, as well as the 1 kb region upstream of the initiation methionine (ATG), were amplified using previously described primers and conditions.[8] Following purification of the PCR products, sequencing reactions were performed using a previously described protocol and then analyzed on an ABI-3100 Genetic Analyzer (Applied Biosystems).[8] Sequences for the 1 kb region upstream of translational start site were compared to the *ZEB1* RefSeqGene sequence (GenBank accession number NG_017048.1). Based on the annotation of *ZEB1* transcript NM_030751.5, the promoter region was defined as the sequences upstream of the transcriptional start site (c.-452) and the 5’ UTR was defined as the region between the transcriptional start site (c.-452) and the translational start site (c.1). Coding region nucleotide sequences (including the donor and acceptor splice sites) were read manually by comparison to the *ZEB1* cDNA sequences (GenBank accession number NM_030751 and NM_001128128.2). The description of the identified sequence variants adhered to the Human Genome Variation Society (HGVS) nomenclature guidelines.

### *OVOL2*—Polymerase chain reaction (PCR) and Sanger sequencing

Each of the four *OVOL2* coding exons and the 1.8kb region upstream of the *OVOL2* translational start site (containing both the 5’ UTR and promoter) were amplified and screened using previously described primer sequences as well as custom designed primers (S1 Table).[25] Based on Genbank *OVOL2* transcript NM_021220.3, the promoter region was defined as the sequences upstream of the transcriptional start site (c.-248) and the 5’ UTR was defined as the region between the transcriptional start site (c.-248) and the translational start site (c.1). PCR reactions were performed using GoTaq^®^ Green Master Mix (Promega, Madison, WI), 240 nM of each forward and reverse primer and 20–40 ng of genomic DNA. Thermal cycling was performed using a C1000 Touch Thermal Cycler (Bio-Rad, Hercules, CA). For the promoter and 5’ UTR screening, reactions were cycled with the following program: initialization at 95°C for 3 minutes; 38 cycles of denaturing, annealing, and extension at 95°C for 25 seconds, 60°C for 25 seconds, 72° for 45 seconds, respectively; and final elongation at 72°C for 10 minutes. For the *OVOL2* coding region screening, reactions were cycled with the following program: denaturation at 95°C for 3 minutes; 38 cycles of annealing and extension at 95°C for 25 seconds, 52°C for 25 seconds, 72° for 30 seconds; and elongation at 72°C for 10 minutes. Prior to sequencing, 15–30 ng of each amplicon was purified by treatment with 5 units of Exonuclease I and 0.5 units of Shrimp Alkaline Phosphatase (USB Corp., Cleveland, OH), followed by incubation at 37°C for 15 minutes and inactivation at 80°C for 15 minutes. Sanger sequencing of the purified PCR template was performed by Laragen Sequencing & Genotyping (Laragen Inc., Culver City, CA).

### CNV analysis in PPCD1 and PPCD3 loci

Genomic DNA samples from eight PPCD probands in whom a *ZEB1* mutation was not identified were submitted to the UCLA Clinical Microarray Core for array comparative genome hybridization (aCGH) to identify copy number variation (CNV) using a custom Agilent 8x60K array (Agilent Technologies, Inc., Santa Clara, CA). The custom array was designed for the interrogation of: a 16.7 Mb region (hg19: 17.3 Mb– 34.0 Mb) on chromosome 20 encompassing the refined PPCD1 interval and approximately 2.7Mb (0.54 Mb 5’ and 2.17 Mb 3’) of flanking sequence; and a 5.2 Mb region (hg19: 29.1Mb– 34.3 Mb) on chromosome 10 containing the ZEB1 gene (31.6 Mb– 31.8 Mb) and 5 Mb (2.5 Mb 5’ and 2.5 Mb 3’) of flanking sequence.[18] The array utilized 52,828 oligonucleotide probes spread over the two loci. Data analysis was performed using the Agilent CytoGenomics 3.0 software. The raw data files are available from the GEO DataSets database (accession number GSE84940; National Center for Biotechnology Information [NCBI], Bethesda, MD, USA).

### Dual-luciferase *OVOL2* promoter activity assay

Immortalized human corneal endothelial cells (HCEnC-21T) were seeded at 50% confluency into 24-well plates. The following day, the cells were transfected using Lipofectamine^®^ LTX (Life Technologies) according to the manufacturer’s recommendations. To test the impact of the *OVOL2* c.-307T>C variant on promoter activity, HCEnC-21T cells were transfected with 250 ng of either a *OVOL2 P* ^WT^, *OVOL2 P* ^c.-307T>C^, or empty (pGL3-Basic) promoter luciferase construct (generously provided by Dr. Alison J. Hardcastle of the University College London).[25] To measure differences in transfection efficiency between wells, 250 ng of pGL4.75[hRluc_CMV] (Promega) was added to each transfection for a total of 500 ng of plasmid DNA per transfection. Forty-eight hours post-transfection, cells were lysed with 100 μL of Passive Lysis Buffer (Promega). Protein levels from cell lysates were quantified using the Pierce^™^ BCA Protein Assay Kit (Life Technologies) and FilterMax F5 microplate reader (Molecular Devices, Sunnyvale, CA). Promoter activity was measured using the Dual-luciferase^®^ Reporter Assay System (Promega) according to manufacturer’s instructions. Each trial was normalized to Renilla firefly luminescence in order to account for variability in transfection efficiency.

### Statistical analyses

The probability and corresponding 95% confidence interval of the identified variant, c.-1409G>C, found within the screened probands was estimated based on the observed frequency in the probands and from the observed frequency of same variant in the population assuming binomial distribution of allele frequencies.

## Results

### Screening of *ZEB1* coding, 5’UTR, and promoter regions in PPCD probands

We have previously reported the absence of presumed pathogenic variants in the non-coding 1 kb region (containing both the 5’ UTR and promoter) upstream of the *ZEB1* translational start site in 31 PPCD probands without a *ZEB1* coding region mutation.[8] After six additional PPCD probands without a *ZEB1* coding region mutation were identified, we screened the 1 kb upstream of the initiation methionine in these six individuals. The known single nucleotide polymorphism (SNP) c.-803G>C (rs3737180; minor allele frequency (MAF) 0.316) was identified in the heterozygous state in two individuals, one of whom also demonstrated a second known SNP (c.-933T>G (rs3737179; MAF 0.316)) in the heterozygous state. Of the 37 DNA samples from these individuals without a *ZEB1* promoter, 5’ UTR, or coding mutation, 27 had sufficient DNA to analyze the 1.8kb region upstream of the *OVOL2* translational start site.

### Exclusion of pathogenic *OVOL2* coding, 5’UTR, and promoter region variants in genetically unresolved PPCD probands

Of the 27 probands who had sufficient DNA to perform screening of the *OVOL2*, one proband (P1) is a member of the family that we mapped to the PPCD1 locus and in which we identified a *OVOL2* promoter variant (c.-307T>C) that segregated with the affected phenotype.[18, 24] Screening of the 1.8 kb region upstream of the translational start site of *OVOL2* in the remaining 26 PPCD probands in whom a *ZEB1* truncating mutation was not identified revealed five different heterozygous variants in eight probands, while the remaining 18 probands did not demonstrate any heterozygous variants within either the *OVOL2* promoter or 5’ UTR (Table 1). Of the five identified variants, only one, c.-61G>A, was either novel or rare (MAF ≤ 0.01). However, the c.-61G>A variant, identified in a single proband (P3), was not identified in his affected father, indicating that it was not causative of PPCD. The only variant identified in more than one proband, c.-1409G>C, has a global MAF of 0.0541. Although the observed frequency of the c.-1409G>C variant in the 27 probands who had sufficient DNA to perform screening of the *OVOL2* promoter (6/54 chromosomes corresponding to an observed MAF of 0.111) was double the global MAF of 0.0541, the difference was not statistically significant (p = 0.064). Given this, and as the global minor allele frequency is much higher than the population prevalence of PPCD, the c.-1409G>C variant is very unlikely to be pathogenic. Screening of each of the four *OVOL2* coding exons in the 27 probands revealed only one heterozygous variant (c.327C>A), which was identified in three probands. As the c.327C>A variant has a MAF of 0.0403, much higher than the population prevalence of PPCD, this variant is also very unlikely to be pathogenic.

**Table 1:** *OVOL2* promoter and 5’ UTR variants identified in 27 probands with PPCD.

Proband ID	# of Probands	Identified Variant	Gene Region	Heterozygosity	RS number	MAF
P1*	1	c.-307T>C	Promoter	Heterozygous	rs869320629	N/A
**P2**	1	c.-1388C>G	Promoter	Heterozygous	rs73252038	0.0323
		c.-186G>A	5’ UTR	Heterozygous	rs73252036	0.0313
		c.-72G>A	5’ UTR	Heterozygous	rs538188467	0.0142
**P3**	1	c.-61G>A**	5’ UTR	Heterozygous	Novel	N/A
**P4 -P9**	6	c.-1409G>C	Promoter	Heterozygous	rs41276412	0.0541
**P10-P27**	18	None identified	-	-	-	-

* Proband belongs to a family previously mapped to the PPCD1 locus;

** Variant was not present in the proband's affected parent

### Identification and segregation of *OVOL2* promoter variant c.-307T>C in the family originally mapped to the PPCD1 locus on chromosome 20

**Fig 1:**
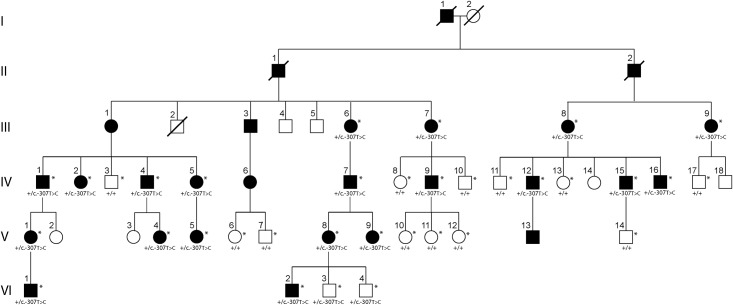
Pedigree of the original family mapped to the PPCD1 locus. Females are represented by circles, males by squares. Affected individuals are shown with filled symbols and unaffected individuals are shown with open symbols. Diagonal lines across symbols indicate individuals who are deceased. An asterisk (*) indicates individuals who underwent *OVOL2* promoter screening by Sanger sequencing.

The 1.8 kb region upstream of the translational start site of *OVOL2* was screened in affected and unaffected members of the family with PPCD that was the first to be mapped to the PPCD1 locus on chromosome 20 (Fig 1).[13] Within the promoter region, the c.-307T>C variant was identified in each of the 20 affected family members and was not identified in 12 of the 14 unaffected members who were screened (Fig 1).

### No copy number variation identified in PPCD1 or PPCD3 loci

CNV analysis within the PPCD1 and PPCD3 loci was performed in eight of the 27 PPCD probands without a *ZEB1* or *OVOL2* coding, 5’ UTR, or promoter region mutation to evaluate for the presence of a potentially pathogenic structural variation. In the PPCD1 locus, 11 CNVs (4 gains and 7 losses) were identified in five of the eight probands, ranging in size from 134 to 14,007 base pairs (S2 Table). Three of the 11 CNVs involved protein-coding genes: two overlapping CNV gains in intron 1 of *XRN2*, with one gain present in one (1/8) proband, and the second gain present in three (3/8) probands; and a loss in intron 2 of *PIGU*, which was identified in three (3/8) probands. There were no identified CNVs within *OVOL2*. In the PPCD3 locus, CNV were identified in eight genomic regions, although none involved *ZEB1*.

### *OVOL2* promoter variant c.-307T>C variant increases *OVOL2* promoter activity

**Fig 2:**
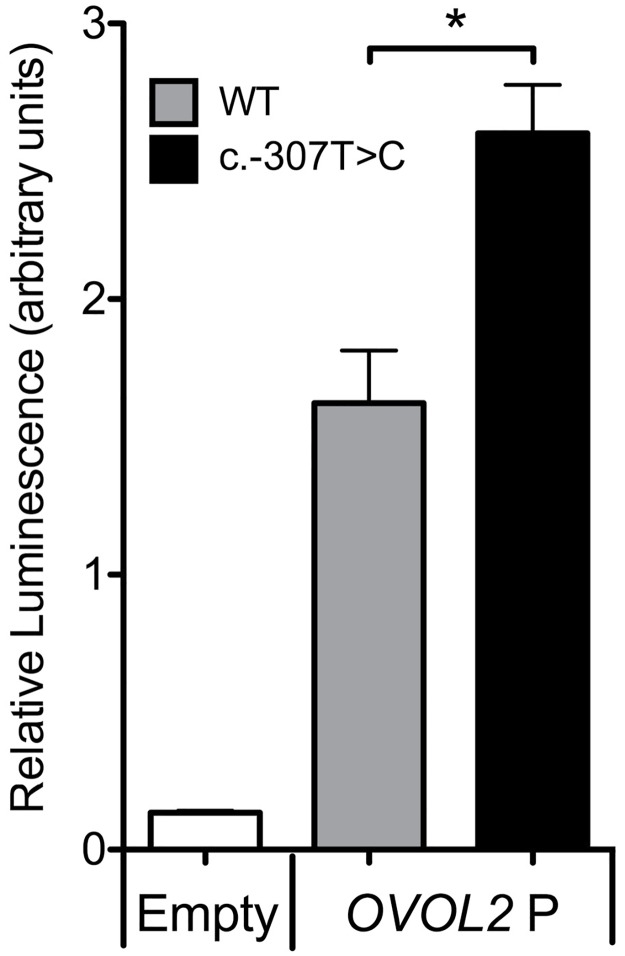
The c.-307T>C variant increases *OVOL2* promoter activity. *OVOL2* promoter constructs containing either wild type (*OVOL2 P* ^*WT*^) or mutant (*OVOL2 P* ^*c*.*-307T*>C^) promoter sequences were transfected into HCEnC-21T cells and the relative luciferase activities of each construct was measured. The *OVOL2 P* ^*c*.*-307T*>C^ promoter construct produced significantly higher levels of luminescence compared to the *OVOL2 P* ^*WT*^ construct. (* p value < 0.05, n = 3, error bars = SEM).

Dual-luciferase reporter assays were performed to determine the effect of the c.-307T>C variant on *OVOL2* promoter activity in human corneal endothelial cells. Immortalized human corneal endothelial cells, HCEnC21T, were transfected with a luciferase reporter construct containing either no promoter (empty), an *OVOL2* wild-type promoter (*OVOL2 P* ^WT^), or an *OVOL2* promoter harboring the c.-307T>C mutation (*OVOL2 P* ^c.-307T>C^). While both *OVOL2 P* ^WT^ and *OVOL2 P* ^c.-307T>C^ were able to drive the expression of luciferase, *OVOL2 P* ^c.-307T>C^ produced significantly higher levels of luciferase expression when compared to the *OVOL2 P* ^WT^ (Fig 2).

## Discussion

The recent identification of *OVOL2* promoter mutations associated with PPCD by Davidson and colleagues as well as our group has led us to screen the *OVOL2* 5’ UTR and promoter region in the family in which linkage analysis established the PPCD1 locus on chromosome 20 and in an additional 26 probands in whom a *ZEB1* truncating mutation was not identified.[24, 25] We identified the same c.-307T>C variant within affected members of the family that Heon and colleagues mapped to the PPCD1 locus as in the family that we subsequently mapped to an overlapping interval.[13, 18] Although the c.-307T>C was found in two unaffected family members (VI-3 and VI-4), these two individuals were first evaluated in their twenties and were not available for re-examination. Therefore, their affected status cannot be confirmed. In this pedigree, the average age at the time of diagnosis of PPCD was 25 years, with a range between 4 and 40 years.[13] Given that the c.-307T>C variant segregates with the affected phenotype in the other 32 affected and unaffected individuals screened, as well as in the 29 individuals in our PPCD1-linked family, the overwhelming likelihood is that these two individuals are in fact affected.[24] This variant has now been demonstrated to segregate with the affected phenotype in three unrelated families that have all been mapped to the PPCD1 locus. Given this, as well as the fact that three other variants in the *OVOL2* promoter have been shown to segregate with the affected phenotype in two other families with PPCD (including the fourth family previously mapped to the PPCD1 locus) and one family initially diagnosed with autosomal dominant CHED that was mapped to the region on chromosome 20 containing *OVOL2*, promoter mutations in *OVOL2* have been convincingly associated with PPCD1.[24, 25]

Our failure to identify a presumed pathogenic *OVOL2* or *ZEB1* coding region or promoter mutation or CNV involving either gene in 26 PPCD probands indicates that other genes may be associated with PPCD. In the PPCD mouse, a chromosomal inversion flanked by deletions involving *Csrp2bp* and *Dzank1* has been identified in the portion of chromosome 2 that is syntenic to the human PPCD1 locus.[28, 29] The investigators reported the upregulation of two *Csrp2bp* fusion transcripts and *Ovol2*, which is located 37 kb from *Csrp2bp* and 66 kb from the breakpoint of the chromosomal inversion. While it is possible that the identified genetic rearrangement leads to PPCD1 in the mouse through upregulation of *Ovol2* expression, as is the case in humans, the authors provide compelling evidence for the disruption of *Csrp2bp* as being causative for PPCD1 in the mouse.[28, 29] However, we did not detect CNV involving the human ortholog *CSRP2BP* in any of the 8 probands analyzed for CNV. Although identification of other affected pedigrees of sufficient size to perform whole exome sequencing and CNV analysis to identify additional genetic loci should be performed, we also plan to perform and encourage other investigators to perform sequencing of presumed *OVOL2* and *ZEB1* regulatory sequences to identify other potentially pathogenic sequence variants.

Additional studies will also need to be performed to elucidate the role of OVOL2 in the corneal endothelium, and to determine whether OVOL2 acts to repress the expression of *ZEB1*, as it does in corneal epithelial cells. OVOL2 maintains the epithelial cell state by repressing mesenchymal genes such as *ZEB1*.[27] When cotransduced with *PAX6*, OVOL2 induces the transcriptional profile of corneal epithelial cells in fibroblasts, suggesting that OVOL2 may serve as a master regulator of the epithelial cell state.[27] In contrast, the roles, if any, that OVOL2 plays in corneal endothelial cell differentiation, function, and dysfunction are virtually unstudied. Here, we demonstrate that the c.-307T>C variant in *OVOL2* results in enhanced promoter activity in corneal endothelial cells compared to the wild-type sequence. As it has been previously reported that OVOL2 represses *ZEB1* expression, our promoter activity results are consistent with the hypothesis that PPCD-associated *OVOL2* promoter mutations lead to PPCD by causing OVOL2 overexpression and subsequent ZEB1 insufficiency.[26, 27] *In silico* analyses have predicted PPCD-associated *OVOL2* promoter mutations cause a gain or loss of putative transcription factor binding sites, potentially leading to *OVOL2* dysregulation.[24, 25] Further investigation into the effects of PPCD-associated *OVOL2* and *ZEB1* mutations on the expression of these transcription factors, the endothelial transcriptome and corneal endothelial morphology and function are ongoing, and are anticipated to clarify whether both PPCD1 and PPCD3 share a common molecular pathway.

## Supporting Information

S1 Table Primers used for *OVOL2* promoter, 5’ UTR, and exon screening. (XLSX)

S2 Table Copy number variants detected by aCGH in 8 probands with posterior polymorphous corneal dystrophy. (XLSX)
